# Pharmacotherapy for Obstructive Sleep Apnea: From Pathophysiology to Emerging Treatments

**DOI:** 10.3390/jcm15166473

**Published:** 2026-08-21

**Authors:** Ruobing Zhou, Ge Yin, Yun Zhu, Yu Sun

**Affiliations:** 1 Department of Otorhinolaryngology, Union Hospital, Tongji Medical College, Huazhong University of Science and Technology, Wuhan 430022, China; 2 Institute of Otorhinolaryngology, Union Hospital, Tongji Medical College, Huazhong University of Science and Technology, Wuhan 430022, China; 3 Hubei Province Clinical Research Center for Deafness and Vertigo, Wuhan 430022, China; 4 Hubei Province Key Laboratory of Oral and Maxillofacial Development and Regeneration, Wuhan 430022, China

**Keywords:** obstructive sleep apnea (OSA), pharmacotherapy, glucagon-like peptide-1 receptor agonists (GLP-1 RAs), tirzepatide, gene therapy, combination therapy

## Abstract

**Background/Objectives**: Obstructive sleep apnea (OSA) is a highly prevalent sleep-related breathing disorder caused by recurrent upper-airway collapse during sleep, leading to intermittent hypoxia, sleep fragmentation, and excessive daytime sleepiness. Although continuous positive airway pressure (CPAP) remains the first-line therapy, long-term adherence is often suboptimal, underscoring the need for more tolerable and flexible treatment options. This review aims to summarize the pathophysiological rationale for pharmacotherapy in OSA and to discuss recent developments in drug-based interventions. **Methods**: We conducted a narrative review of the literature on pharmacological interventions for OSA, with a systematic search of PubMed, Embase, Cochrane Library, and ClinicalTrials.gov up to 4 August 2026. We included randomised controlled trials, observational studies, meta-analyses, and mechanistic human or animal studies that reported relevant sleep and respiratory outcomes, and graded evidence according to the principles of GRADE framework. **Results**: Several drug classes have shown promise: agents that increase upper-airway dilator muscle activity, respiratory stabilizers that reduce loop gain, medications that raise the arousal threshold, topical anti-inflammatory drugs for mucosal edema, and systemic metabolic modulators such as glucagon-like peptide-1 receptor agonists and dual incretin receptor agonists. Emerging strategies, including gene therapy directed at the hypoglossal motor system, are also under investigation at the preclinical stage. Moreover, combining pharmacotherapy with CPAP or other devices can produce synergistic benefits, enabling lower device pressures and enhanced patient comfort. **Conclusions**: Pharmacotherapy for OSA is progressively moving from exploratory research towards targeted, phenotype-driven personalized treatment. Future studies should focus on robust patient phenotyping, multi-mechanistic combination regimens, and long-term clinical outcome evaluations to facilitate the integration of drug-based therapies into routine OSA management, particularly in specific subgroups such as those with COMISA.

## 1. Introduction

Obstructive sleep apnea (OSA) is a common sleep-related breathing disorder characterized by recurrent episodes of partial or complete collapse of the upper airway during sleep, resulting in intermittent hypoxia, sleep fragmentation, and excessive daytime sleepiness [1]. OSA has a high global prevalence. According to recent estimates, using an apnea–hypopnea index (AHI) ≥ 5 events per hour as the diagnostic threshold, approximately 936 million adults are affected worldwide, with an estimated 176 million individuals in China, representing the largest patient population globally [2]. The prevalence is particularly high among individuals who are obese, elderly, or male. OSA not only significantly impairs quality of life but is also closely associated with multiple systemic complications, including cardiovascular disease, stroke, diabetes mellitus, and neurocognitive dysfunction, thereby imposing a substantial public health burden [3].

The pathophysiology of OSA is multifactorial and involves upper airway anatomical abnormalities, impaired neuromuscular control, low respiratory arousal threshold, unstable ventilatory control (high loop gain), and obesity-related metabolic disturbances [1,4,5,6,7]. Continuous positive airway pressure (CPAP) therapy remains the first-line treatment for OSA. However, long-term adherence to CPAP therapy is often suboptimal, with reported non-adherence rates ranging from 30% to 80% depending on definitions and populations [8,9], and treatment effectiveness may be limited in certain patient populations [7]. Consequently, pharmacological therapy has emerged as a potential complementary strategy in the management of OSA. Given the recent advances in both deciphering OSA endotypes and the development of mechanism-based pharmacotherapies, this review aims to summarize the pathophysiological basis for pharmacological interventions, outline recent clinical progress in drug-based treatments, discuss emerging combination strategies, and highlight future directions for personalized therapy.

## 2. Methods

We conducted a narrative review of the literature on pharmacological interventions for obstructive sleep apnea. We searched PubMed, Embase, Cochrane Library, and ClinicalTrials.gov from inception to 4 August 2026, using combinations of the following search terms: “obstructive sleep apnea”, “OSA”, “pharmacotherapy”, “drug therapy”, “GLP-1 receptor agonists”, “tirzepatide”, “atomoxetine”, “oxybutynin”, “aroxybutynin”, “reboxetine”, “acetazolamide”, “sultiame”, “eszopiclone”, “zopiclone”, “trazodone”, “intranasal corticosteroids”, “SGLT-2 inhibitors”, “gene therapy”, and “chemogenetics”. We also manually reviewed reference lists of included articles and relevant systematic reviews. We included randomised controlled trials (RCTs), observational studies (cohort and case–control), meta-analyses, and mechanistic human or animal studies that reported AHI or other sleep-related respiratory outcomes (e.g., oxygen desaturation index, hypoxic burden, Epworth Sleepiness Scale). We excluded case reports, conference abstracts, and non-English articles. Study selection and data extraction were performed by two authors independently, with disagreements resolved by consensus.

For evidence quality assessment, we applied principles from the GRADE (Grading of Recommendations Assessment, Development and Evaluation) framework, adapted for the narrative scope of this review. Evidence was graded as follows: high (consistent findings from multiple well-designed RCTs or a single large RCT with robust effect estimates); moderate (limited RCTs, or well-designed observational studies with large effect sizes); low (observational studies with inconsistency or risk of bias, or evidence derived primarily from post-hoc or subgroup analyses); and very low/preclinical (animal experiments, mechanistic studies, or case series, with no human trials available). This grading is applied throughout the text to help readers interpret the strength of the evidence underlying each therapeutic approach.

## 3. Pathophysiological Basis of Pharmacological Treatment in OSA

Obstructive sleep apnea was long considered primarily an anatomical disorder of the upper airway, with craniofacial bony structures, soft tissue volumes, and pharyngeal length being the main determinants of airway collapsibility [4,5]. However, the poor long-term adherence to CPAP and the observation that many patients with similar anatomical severity exhibit widely different AHI prompted a shift toward understanding non-anatomical pathophysiological traits, or “endotypes” [1,6]. Over the past decade, advances in respiratory physiology have identified four major non-anatomical contributors to OSA: impaired pharyngeal dilator muscle responsiveness, low respiratory arousal threshold, unstable ventilatory control (high loop gain), and obesity-driven metabolic and mechanical changes [7,10]. Importantly, each of these endotypes can be targeted by specific pharmacological agents, which has stimulated a wave of drug repurposing and novel compound development.

### 3.1. Impaired Upper Airway Dilator Muscle Function

During sleep, neuromuscular drive to the genioglossus and other pharyngeal dilators falls, allowing airway collapse. The degree of this impairment varies among patients and can be quantified by the reduction in muscle activity from wakefulness to sleep [7]. The genioglossus, the primary upper airway dilator, is innervated by the hypoglossal nerve. Its activity is under strong noradrenergic excitatory control during wakefulness, but this drive falls during sleep, contributing to airway collapse. This noradrenergic dependency provides a direct rationale for pharmacologically enhancing hypoglossal motor output. Experimental animal studies first identified that certain potassium channels expressed in hypoglossal motor neurons suppress genioglossus activity during sleep; local blockade of these channels markedly reactivates the muscle [10,11]. Translating this concept to humans, pharmacological enhancement of noradrenergic and muscarinic signaling has been tested. Norepinephrine reuptake inhibitors (NRIs) and antimuscarinic agents represent the primary drug classes targeting this endotype.

### 3.2. Low Respiratory Arousal Threshold

Some patients awaken from sleep very early in response to minimal airway resistance or mild oxygen desaturation, leading to fragmented sleep without allowing sufficient time for dilator muscle recruitment. This trait, termed low arousal threshold, can be identified using clinical parameters (e.g., low AHI, high nadir oxygen saturation) [6]. Sedative hypnotics that raise the arousal threshold (e.g., trazodone, zopiclone, and eszopiclone) have been evaluated as a strategy to prolong respiratory epochs and improve sleep continuity in this subgroup. Cholinesterase inhibitors (e.g., donepezil) represent another strategy to stabilize sleep by enhancing cholinergic transmission [12].

### 3.3. Unstable Ventilatory Control (High Loop Gain)

A hypersensitive chemoreflex system over-responds to small changes in PaCO_2_, driving periodic breathing and recurrent airway collapse. High loop gain is common in patients with low arousal threshold and can be measured by the loop gain parameter during sleep studies [7]. Carbonic anhydrase inhibitors (acetazolamide, sultiame) induce mild metabolic acidosis, which increases basal ventilatory drive and reduces controller gain, thereby stabilizing breathing. These agents directly target the ventilatory instability that perpetuates apnoeic events in susceptible patients.

### 3.4. Obesity and Metabolic Dysregulation

Obesity contributes to OSA through multiple mechanisms: pharyngeal fat deposition, increased abdominal mass loading, reduced lung volume, and low-grade systemic inflammation. Weight loss is the most effective non-CPAP intervention, but lifestyle modification alone is rarely sustained. The advent of incretin-based therapies—initially developed for diabetes and obesity—has provided a powerful tool to address the obesity component of OSA. Glucagon-like peptide-1 (GLP-1) receptor agonists (liraglutide, semaglutide) produce dose-dependent weight loss and parallel AHI improvements [13]. These metabolic agents directly target a core pathophysiological driver of OSA in obese patients.

## 4. Advances in Pharmacological Treatment

### 4.1. Drugs Targeting Upper Airway Dilator Muscles

Norepinephrine reuptake inhibitors (NRIs) such as desipramine and atomoxetine enhance hypoglossal motor output by increasing synaptic norepinephrine availability, a strategy supported by the noradrenergic control of genioglossus activity. In a randomized trial, desipramine 200 mg improved active pharyngeal critical closing pressure (Pcrit) from −1.9 to −5.2 cmH_2_O and reduced AHI, particularly in patients with poor baseline neuromuscular compensation [14]; however, desipramine is associated with anticholinergic side effects (dry mouth, constipation, sedation) that may limit tolerability.

A four-week trial of atomoxetine 40–80 mg daily (*n* = 20, mild-to-moderate OSA with sleepiness) showed significant improvements in daytime sleepiness, with the Epworth Sleepiness Scale (ESS) decreasing from 15.3 to 10.5 (*p* < 0.01), and the Clinical Global Impression (CGI) score improving from 4.3 to 3.1 (*p* < 0.01). Both ESS and CGI improved in a linear fashion across the treatment weeks. However, there was no significant change in the respiratory disturbance index (RDI). Notably, sleep architecture was affected, with decreased sleep efficiency and reduced percentage of rapid eye movement (REM) sleep, alongside increased percentage of stage 1 sleep, awakenings, and wake after sleep onset [15]. These findings suggest that atomoxetine monotherapy may ameliorate subjective sleepiness but does not reliably improve objective respiratory events, possibly due to its adverse effects on sleep continuity.

Beyond NRIs, preclinical studies have identified potassium channels (inward-rectifying and voltage-gated) in hypoglossal motor neurons as a novel target; local blockade in rats increased genioglossus activity during NREM and REM sleep to 133–300% of wakefulness levels [10]. As these potassium channel blockers have not yet entered clinical trials, their safety profile in humans remains unknown.

Antimuscarinic agents such as aroxybutynin enhance upper airway muscle contraction by blocking M2 muscarinic receptors, countering the sleep-related decline in pharyngeal tone. The combination AD109 (aroxybutynin plus atomoxetine) has demonstrated dose-dependent reductions in AHI in patients with mild-to-moderate OSA. In the MARIPOSA study, a four-week phase 2b randomised, double-blind, placebo-controlled trial, 211 patients with mild-to-severe OSA were enrolled. In the AD109 2.5/75 mg group, the median AHI decreased from 20.5 events per hour at baseline to 10.8 events per hour, representing a 47.1% reduction (placebo-subtracted difference −47.1%, 95% CI −61.2 to −27.9; *p* < 0.0001). Furthermore, 41% of patients achieved a post-treatment AHI below 10 events per hour, and significant improvements were observed in daytime fatigue scores (*p* < 0.05) [16]. However, it should be noted that the trial compared combination therapy against placebo, and head-to-head comparisons of the combination versus atomoxetine monotherapy were not explicitly reported; therefore, the incremental benefit of adding aroxybutynin over atomoxetine alone remains to be formally established.

Another one-week randomised, double-blind, placebo-controlled crossover trial evaluated reboxetine combined with oxybutynin in 16 patients with OSA (median age 57 years, BMI 30 (26–36) kg/m^2^). Compared with placebo (median AHI reduction 6%), the combination therapy produced a significant median AHI reduction from 49 events per hour at baseline to 18 events per hour (59% median reduction, *p* < 0.001). The response rate (defined as AHI reduction ≥ 50%) for reboxetine plus oxybutynin was 81% versus 13% for placebo (*p* < 0.001). Although ESS scores were not significantly lowered, psychomotor vigilance task (PVT) median reaction time improved significantly from 250 ms at baseline to 223 ms on active treatment versus 264 ms on placebo (*p* < 0.001). Home oximetry also demonstrated acute and sustained improvement in the oxygen desaturation index with the combination. The treatment was well tolerated, suggesting that noradrenergic–antimuscarinic combination therapy represents a promising pharmacological strategy for OSA [17].

### 4.2. Drugs Targeting High Loop Gain (Unstable Ventilatory Control)

Carbonic anhydrase inhibition represents a validated approach to stabilize ventilatory control in patients with high loop gain. Acetazolamide, a conventional carbonic anhydrase inhibitor, has been shown to reduce loop gain and lower AHI by approximately 40% in patients with a high-loop-gain phenotype, with sustained benefit during long-term therapy [18,19]. However, its utility is limited by dose-related side effects including paresthesia, metallic taste, and polyuria.

More recently, the novel carbonic anhydrase inhibitor sultiame has been systematically evaluated in the phase 2 FLOW trial. In this multicentre, randomised, double-blind, placebo-controlled, dose-finding trial, 535 patients were screened and 298 were randomly assigned to placebo (*n* = 75), sultiame 100 mg (*n* = 74), 200 mg (*n* = 74), or 300 mg (*n* = 75) for 15 weeks; 240 patients completed the full treatment period. In the full analysis set, the placebo-subtracted relative AHI_3a_ adjusted mean changes at week 15 were −16.4% (95% CI −31.3 to −1.4; *p* = 0.032) for 100 mg, −30.2% (95% CI −45.4 to −15.1; *p* < 0.0001) for 200 mg, and −34.6% (95% CI −49.1 to −20.0; *p* < 0.0001) for 300 mg, demonstrating dose-dependent efficacy. Adverse events increased dose-dependently: 46 (61%) of 75 patients in the placebo group, 54 (73%) of 74 in the 100 mg group, 62 (84%) of 74 in the 200 mg group, and 68 (91%) of 75 in the 300 mg group. The most common adverse event was paraesthesia, reported in 7 (9%) of placebo, 16 (22%) of 100 mg, 32 (43%) of 200 mg, and 43 (57%) of 300 mg recipients. Headache was also more frequent in the 200 mg (16%) and 300 mg (15%) groups compared with placebo (8%). The 200 mg dose offered the best balance of efficacy and safety [20]. Unlike acetazolamide, sultiame may also improve upper airway muscle tone through carbonic anhydrase inhibition in pharyngeal muscles, suggesting a dual mechanism that could be particularly beneficial in patients with combined loop gain and muscle dysfunction, although this hypothesis requires further mechanistic validation in humans.

### 4.3. Drugs Targeting Low Respiratory Arousal Threshold

Low respiratory arousal threshold is a distinct endotype characterized by premature awakening in response to minimal airway resistance or mild oxygen desaturation, leading to sleep fragmentation without sufficient time for dilator muscle recruitment. For these patients, sedative-hypnotics (trazodone, zopiclone, eszopiclone) raise the arousal threshold, allowing more time for dilator muscle recruitment. Trazodone 100 mg increased the arousal threshold during NREM sleep without worsening upper airway collapsibility [21]; zopiclone similarly increased arousal threshold without impairing genioglossus activity [22].

A systematic review and meta-analysis of 17 studies comprising 2099 patients evaluated the effect of non-benzodiazepine sedative hypnotics (NBSH) on respiratory parameters during sleep. The mean AHI in the NBSH group was 13.17 ± 16.27 versus 15.94 ± 19.31 in the control group (mean difference 2.77, 95% CI 1.463–4.076). Subgroup analyses revealed differential effects across agents: four studies (362 patients) comparing eszopiclone with placebo demonstrated a significant AHI reduction (mean difference −5.73 events/h, 95% CI −8.90 to −2.57), whereas six studies (100 patients) comparing zolpidem with placebo or no medication showed no significant effect (mean difference −0.61 events/h, 95% CI −1.94 to 0.71, *p* = 0.36). Two large studies (979 patients) comparing both zolpidem and eszopiclone to no medication found an AHI mean difference of −1.66 events/h (95% CI −5.87 to 2.55) [23]. These findings suggest that eszopiclone may offer modest AHI improvement, although the clinical significance of this effect warrants further investigation. Cholinesterase inhibitors (e.g., donepezil) also increase the arousal threshold via enhanced cholinergic transmission, but they have not consistently reduced AHI [24]. Common side effects of sedatives include residual daytime drowsiness and risk of dependence, which require careful patient selection, particularly in patients with comorbid insomnia.

In clinical practice, a substantial proportion of patients with OSA also have comorbid insomnia, a condition termed COMISA (co-morbid insomnia and sleep apnoea). In these patients, the use of sedative-hypnotics requires particular caution. While these agents may improve sleep continuity by raising the arousal threshold, they carry a heightened risk of worsening respiratory events if upper airway tone is further compromised, especially in patients with significant anatomical collapsibility. Conversely, noradrenergic agents (e.g., atomoxetine, reboxetine) may improve pharyngeal muscle tone but can exacerbate insomnia due to their wake-promoting effects. Current practice therefore favours a multimodal approach for COMISA, with cognitive behavioural therapy for insomnia (CBT-I) as the first-line non-pharmacological intervention, often combined with CPAP; pharmacological agents are reserved for selected patients after careful phenotyping, starting at low doses with close monitoring of respiratory parameters [25,26].

### 4.4. Drugs Targeting Obesity and Metabolic Dysfunction

As detailed in the pathophysiological section, obesity drives OSA through multiple mechanisms including pharyngeal fat deposition, reduced lung volume, and systemic inflammation [7]. Weight reduction therefore represents a fundamental therapeutic approach that addresses the root cause of obesity-driven OSA. However, lifestyle modification alone achieves only modest and poorly sustained weight loss [27], and bariatric surgery, though effective, is invasive and not suitable for all patients [28]. Over the past decade, highly effective pharmacotherapies for obesity have emerged, offering new opportunities to target the metabolic drivers of OSA.

All currently available weight loss pharmacotherapies for OSA, including GLP-1 receptor agonists and dual/triple incretin receptor agonists, exert their primary effects through weight reduction. While tirzepatide has demonstrated greater AHI reductions than earlier GLP-1 agonists, mediation analyses confirm that weight loss accounts for a substantial proportion of its benefit. These agents are best understood as metabolic modulators whose benefits are largely mediated by weight loss, with potential additional mechanisms (e.g., anti-inflammatory effects, improved upper airway fat deposition) that remain to be fully characterised.

#### 4.4.1. Incretin-Based Therapies: From GLP-1 Receptor Agonists to Tirzepatide

Glucagon-like peptide-1 (GLP-1) receptor agonists, including liraglutide and semaglutide, promote weight loss through centrally mediated appetite suppression, delayed gastric emptying, and improved glycemic control [13]. Several clinical trials have shown that these agents reduce the apnea–hypopnea index (AHI) in parallel with the magnitude of weight reduction, but none have received regulatory approval specifically for the treatment of OSA, and their effect on OSA is considered to be mediated primarily by weight loss [29]. Common gastrointestinal side effects (nausea, vomiting, diarrhea) are usually mild to moderate and tend to diminish over time [13].

In December 2024, the U.S. Food and Drug Administration (FDA) approved tirzepatide injection as the first pharmacotherapy specifically indicated for moderate-to-severe OSA in adults with obesity [30]. Tirzepatide is a once-weekly, dual agonist of the glucose-dependent insulinotropic polypeptide (GIP) and GLP-1 receptors, which produces more pronounced weight loss than GLP-1 monotherapy [13,30]. The landmark SURMOUNT-OSA trial program comprised two 52-week, randomized, double-blind, placebo-controlled phase 3 studies. In Study 1 (patients with moderate-to-severe OSA and obesity not using positive airway pressure [PAP] therapy), tirzepatide reduced AHI by approximately 55% from baseline (least squares mean change −25.3 events/h; placebo-subtracted difference −20.0 events/h, 95% CI −25.8 to −14.2; *p* < 0.001), with 43.0% of participants achieving disease remission (AHI < 5 events/h or AHI 5–14 with ESS ≤ 10). In Study 2 (patients on PAP therapy), the mean AHI reduction was approximately 62.8% (placebo-subtracted difference −23.8 events/h, 95% CI −29.6 to −17.9; *p* < 0.001), with 51.5% achieving disease remission. Across both studies, mean body weight reduction ranged from 15% to 20% after 52 weeks of treatment [30].

Beyond AHI improvement, tirzepatide produced substantial cardiometabolic benefits. A prespecified secondary analysis of SURMOUNT-OSA demonstrated significant improvements in systolic blood pressure, glycemic control (including homeostatic model assessment for insulin resistance, HOMA-IR), high-sensitivity C-reactive protein (hs-CRP), triglycerides, and other inflammatory and lipid parameters [31]. Mediation analysis indicated that while weight reduction contributed substantially to these benefits, improvements in OSA metrics (AHI and sleep apnea-specific hypoxic burden) independently mediated reductions in hs-CRP, HOMA-IR, and triglycerides, suggesting that treating both obesity and sleep-disordered breathing is necessary to optimize cardiometabolic outcomes in this population [31].

Time-course analyses from SURMOUNT-OSA showed that tirzepatide-associated improvements in peripheral AHI became significant versus baseline as early as week 4, but the estimated treatment difference compared to placebo did not reach statistical significance until week 20 for both AHI and sleep apnea-specific hypoxic burden [32]. The magnitude of AHI improvement was closely correlated with achieved weight reduction, although additional weight-independent effects cannot be excluded and warrant further investigation [31].

Tirzepatide is indicated for adults with obesity (BMI ≥ 30 kg/m^2^) and moderate-to-severe OSA. It provides a valuable therapeutic option that can be used as an alternative or adjunct to conventional therapies, particularly for patients who have difficulty adhering to PAP or who prefer a non-device approach. However, it is not intended to replace PAP in all patients, and treatment decisions should be individualised based on patient phenotype, preferences, and comorbidities. The most common adverse events with tirzepatide were gastrointestinal (nausea, diarrhea, vomiting), generally mild to moderate in severity and more frequent during dose escalation. Serious adverse events were uncommon and occurred at similar rates to placebo [31].

#### 4.4.2. Emerging Incretin-Based and Metabolic Therapies

Several additional incretin-based agents are under active investigation for OSA. Retatrutide, a triple agonist targeting GIP, GLP-1, and glucagon receptors, has demonstrated substantial weight-lowering potential (up to 24% weight reduction at 48 weeks) in phase 2 obesity trials [33]. The TRIUMPH registrational clinical program, comprising four phase 3 trials (TRIUMPH-1 to TRIUMPH-4), is now evaluating retatrutide concurrently for the treatment of obesity, OSA, and knee osteoarthritis in over 5800 participants, with change in AHI as a primary endpoint in the nested OSA protocols [34].

Maridebart cafraglutide (formerly AMG 133), a long-acting peptide-antibody conjugate administered monthly or less frequently, is a GIP receptor antagonist combined with GLP-1 receptor agonism [35]. Two phase 3 trials are ongoing: one in adults with OSA receiving PAP therapy (NCT07225686) and another in those not on PAP therapy (NCT07226765), both with 52-week treatment periods and AHI as the primary outcome. Similarly, HRS9531, a GIP/GLP-1 dual agonist developed in China, has entered phase 3 testing for OSA with obesity, with two parallel trials (NCT06974851 for patients not on PAP therapy and NCT06994650 for those on PAP therapy).

#### 4.4.3. Sodium-Glucose Cotransporter-2 (SGLT-2) Inhibitors: An Emerging Class

Beyond incretin-based drugs, sodium-glucose cotransporter-2 (SGLT-2) inhibitors—developed primarily for type 2 diabetes mellitus (T2DM) and heart failure—have recently attracted attention for their potential benefits in OSA [36]. Several meta-analyses have evaluated the impact of SGLT-2 inhibitors on sleep-disordered breathing. A meta-analysis of two randomized controlled trials showed that SGLT-2 inhibitors significantly reduced the incidence of OSA by 53% (RR = 0.53, 95% CI: 0.35–0.80, *p* = 0.003) [37]. A larger systematic review and meta-analysis (including nine studies, six RCTs and three non-RCTs) demonstrated that SGLT-2 inhibitors were superior to control interventions (other hypoglycemic drugs) in reducing AHI (mean difference [MD] = −12.57, 95% CI: −21.47 to −3.66, *p* = 0.006) and increasing the lowest oxygen saturation (lowest SpO_2_). Furthermore, patients with T2DM receiving SGLT-2 inhibitors showed a 50% relative risk reduction for incident OSA compared with placebo [36]. Another meta-analysis of four studies involving 686 patients found a statistically significant reduction in AHI (MD = −5.52, 95% CI: −9.72 to −1.32, *p* = 0.01), oxygen desaturation index (MD = −3.16, *p* = 0.004), and BMI (MD = −1.29, *p* = 0.005). However, SGLT-2 inhibitors failed to significantly improve daytime sleepiness (Epworth Sleepiness Scale), hemoglobin A1c, blood pressure, or lipid profiles [38]. The adverse event rate was comparable between SGLT-2 inhibitor and control groups [36].

Important caveat: The evidence summarised above derives primarily from post-hoc or subgroup analyses of diabetes and heart failure trials, rather than from dedicated OSA trials using polysomnography as the primary endpoint. The effect sizes vary considerably across meta-analyses, likely reflecting differences in study populations (whether patients had T2DM, baseline OSA severity, comparator agents, and diagnostic criteria for OSA). The quality of evidence is rated as low to moderate due to inconsistency and indirectness (GRADE assessment), and further large-scale, well-controlled trials specifically designed for OSA are needed to confirm efficacy and safety [38]. The mechanisms underlying SGLT-2 inhibitor effects on OSA remain speculative but may involve weight reduction, improvement in fluid overload (reducing overnight rostral fluid shift, a contributor to pharyngeal narrowing), attenuation of systemic inflammation, and modulation of sympathetic nervous system activity. The ADIPOSA study (NCT06878066), currently underway, aims to elucidate these mechanisms and provide evidence for future trials. For now, these findings should be considered exploratory and hypothesis generating; they are not yet sufficient to change clinical practice.

### 4.5. Local Anti-Inflammatory Therapy

Upper airway mucosal oedema from inflammation contributes to airway narrowing in OSA. In children, intranasal corticosteroids are effective for mild-to-moderate paediatric OSA associated with adenoid hypertrophy [39,40]. In adults, the evidence is more nuanced and population-dependent. Several randomised controlled trials have evaluated intranasal corticosteroids in specific subgroups. In moderate-to-severe OSA with coexisting chronic rhinitis (BMI < 30 kg/m^2^, non-severe oropharyngeal obstruction), fluticasone furoate 110 μg/day for 4 weeks did not significantly improve AHI between groups, but within-group analysis showed significant reductions in AHI, respiratory disturbance index (RDI), non-supine RDI, and sleep quality, with non-supine RDI improvement being significantly greater than placebo (*p* = 0.01) [41]. In patients with allergic rhinitis, intranasal fluticasone reduced AHI from 30.3 to 23.3 (*p* < 0.05) and improved daytime alertness [42]. A meta-analysis pooling data from five RCTs (*n* = 221) showed a small but statistically significant effect on AHI reduction (standardised mean difference −0.95, 95% CI −1.42 to −0.47), although the evidence was limited by heterogeneity and risk of bias across the included studies (I^2^ = 62%) [43]. However, in unselected mild-to-moderate OSA without rhinitis, combined anti-inflammatory therapy (montelukast plus intranasal budesonide) did not significantly reduce AHI, although total sleep time and REM sleep percentage increased significantly [43].

Based on currently available evidence (low to moderate quality), intranasal corticosteroids are not recommended as monotherapy for unselected adult OSA. They may be considered as adjunctive therapy in patients with: (1) refractory nasal congestion or allergic rhinitis that may worsen airway narrowing; (2) moderate-to-severe OSA with coexisting chronic rhinitis who decline or are intolerant to CPAP; or (3) CPAP-induced rhinitis limiting adherence.

### 4.6. Gene Therapy

All strategies described in this section are currently at the preclinical stage, with no human trials reported to date. The following discussion is intended to highlight emerging mechanistic approaches rather than clinically available treatments.

#### 4.6.1. Chemogenetic Strategy Targeting Hypoglossal Motoneurons

Chemogenetics using the Designer Receptor’s Exclusively Activated by Designer Drugs (DREADD) system enables selective activation of hypoglossal motoneurons. Early proof-of-concept studies delivered excitatory DREADD receptors via adeno-associated viral (AAV) vectors into the hypoglossal nucleus, and systemic administration of the ligand chemical clozapine-N-oxide (CNO) significantly increased genioglossus muscle activity and improved upper airway patency [44]. To overcome the translational barriers of direct brainstem injection and CNO-associated toxicity, a more clinically feasible approach was developed using retrograde AAV9 delivery via intralingual injection combined with the novel DREADD ligand JHU37160-dihydrochloride (J60). This technique avoids intracranial surgery and produces a >6-fold increase in genioglossus tonic activity, effectively treating sleep apnea in obese mice [45]. Unlike CNO, J60 does not convert to psychoactive metabolites, substantially improving translational potential.

#### 4.6.2. Optogenetic Strategy Targeting Upper Airway Muscles

Optogenetics enables light-induced activation of upper airway muscles with precise spatiotemporal control. Intralingual delivery of an AAV vector expressing channelrhodopsin under a muscle-specific promoter achieved sufficient opsin expression to restore electromyographic signals and dilate the upper airway [46].

#### 4.6.3. Bioluminescence-Optogenetics Strategy

Bioluminescence-optogenetics (BL-OG) combines genetic precision with non-invasive activation via bioluminescence. A retrograde rAAV2/Retro vector expressing eLMO3 protein was peripherally injected to target the hypoglossal nucleus. After administration of the substrate coelenterazine, bioluminescence activated channelrhodopsin, increasing genioglossus activity and improving apnea index without affecting sleep structure [47]. This strategy requires neither intracranial injection nor external light delivery, representing a significant advance toward clinical translation.

### 4.7. Safety and Tolerability

Across the reviewed agents, safety profiles vary substantially by drug class and must be carefully considered in clinical decision-making.

For noradrenergic and antimuscarinic agents (atomoxetine, reboxetine, oxybutynin, aroxybutynin), the most common adverse effects include insomnia, dry mouth, constipation, and urinary hesitation. In the MARIPOSA trial, adverse events were more frequent with AD109 than placebo but were generally mild to moderate; discontinuation rates due to adverse events were low [16]. Atomoxetine monotherapy was associated with reduced REM sleep and increased awakenings, which may limit its acceptability in patients with pre-existing insomnia [15].

For carbonic anhydrase inhibitors (acetazolamide, sultiame), dose-related paraesthesia is the most frequently reported adverse effect, occurring in up to 57% of patients at the highest sultiame dose (300 mg) in the FLOW trial. Headache, polyuria, and metallic taste are also common. The 200 mg dose of sultiame offered the best balance of efficacy and safety, with a lower incidence of paraesthesia (43%) compared with the 300 mg dose (57%) [20]. Acetazolamide is also associated with metabolic acidosis and renal stone formation with prolonged use.

For sedative-hypnotics (eszopiclone, zopiclone, trazodone), residual daytime drowsiness, cognitive impairment, and risk of dependence are major concerns. Although eszopiclone showed modest AHI improvement in meta-analyses, its use in OSA patients should be restricted to those with confirmed low arousal threshold and without significant baseline hypoxaemia, and should be monitored carefully for worsening of respiratory events or next-day sedation.

For incretin-based therapies (tirzepatide, semaglutide, liraglutide), gastrointestinal adverse events (nausea, vomiting, diarrhoea, constipation) are the most common and are dose-dependent. These are generally mild to moderate, peak during dose escalation, and diminish over time. Serious adverse events are uncommon; in SURMOUNT-OSA, the rates of serious adverse events were similar between tirzepatide and placebo groups [30]. Pancreatitis and gallbladder disease (including cholelithiasis) have been reported with GLP-1 receptor agonists, although absolute risks are low. Long-term safety data beyond one year are still accumulating.

For SGLT-2 inhibitors, the adverse event profile appears favourable, with rates comparable to placebo in meta-analyses. Genitourinary infections and volume depletion are the most commonly reported events, but the evidence in OSA-specific populations remains limited [36].

**Figure 1:**
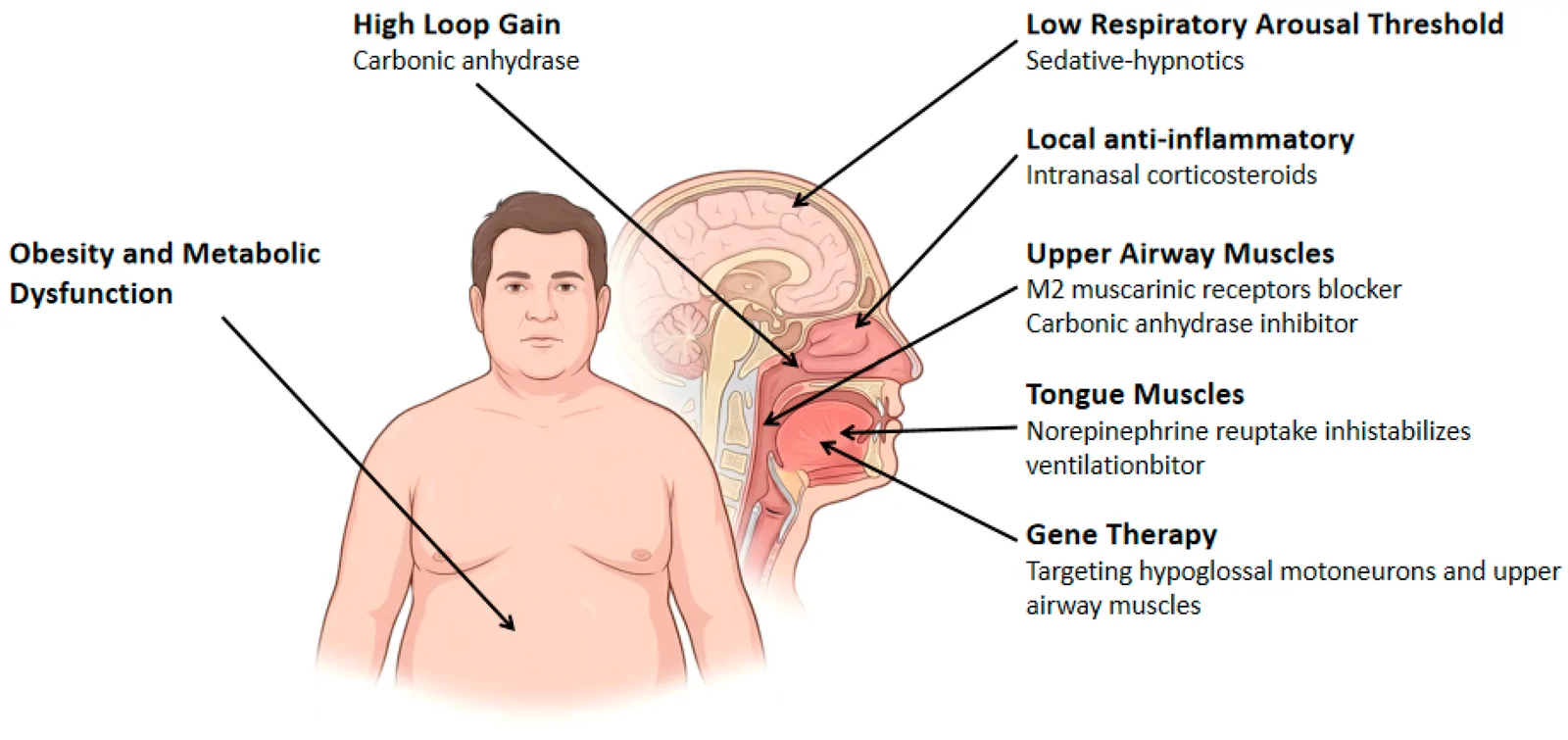
Schematic of drug targets in OSA.

Gene therapy approaches remain preclinical, and safety data in humans are not yet available. Potential risks include immune responses to viral vectors, off-target effects, and the long-term consequences of sustained motoneuron activation (Figure 1).

## 5. Combination Pharmacotherapy Strategies

With the growing recognition that obstructive sleep apnea (OSA) arises from multiple interacting pathophysiological mechanisms, combination therapeutic strategies have increasingly become an important direction in OSA management.

### 5.1. Pharmacotherapy Combined with CPAP

Pharmacological agents may be used to enhance tolerance and adherence to continuous positive airway pressure (CPAP) therapy. For example, the administration of eszopiclone during the initial phase of CPAP treatment has been shown to improve patient adaptation to CPAP and increase long-term adherence [48,49]. A secondary analysis further indicated that this beneficial effect is particularly pronounced in patients with a low arousal threshold [50]. By promoting sleep initiation and reducing discomfort associated with CPAP use during early treatment, eszopiclone may facilitate successful acclimatization to CPAP therapy.

### 5.2. Multi-Mechanism Drug Combinations

Another important approach involves combining pharmacological agents that target different pathophysiological mechanisms of OSA.

The MARIPOSA study evaluated the combination of aroxybutynin and atomoxetine (AD109) as described above. The combination significantly reduced AHI compared with placebo. However, the trial did not include a head-to-head comparison of the combination versus atomoxetine alone; the incremental benefit of adding aroxybutynin therefore remains to be established. Nevertheless, targeting both noradrenergic and muscarinic pathways is a rational strategy to address the multifactorial pathophysiology of upper airway collapse [16].

The reboxetine plus oxybutynin crossover trial similarly demonstrated marked efficacy compared with placebo, with a response rate of 81% and a median AHI reduction of 59%. These findings collectively support the concept that multi-mechanistic pharmacological strategies may offer substantial benefit in appropriately selected patients [17].

### 5.3. Pharmacotherapy Combined with Oral Appliance Therapy

Pharmacological agents may also be combined with oral appliance therapy (OAT) to enhance therapeutic efficacy. Local anti-inflammatory drugs or medications that increase upper airway muscle tone may help optimize the stabilizing effects of oral appliances on upper airway structure, thereby improving airway patency during sleep [51]. Such combination approaches may be particularly beneficial in patients with mild-to-moderate OSA [52]. However, clinical data specifically evaluating pharmacotherapy–OAT combinations remain sparse (Table 1).

## 6. Conclusions

The pharmacological landscape for obstructive sleep apnea has evolved substantially over the past decade, transitioning from exploratory drug repurposing to mechanism-based, endotype-directed therapies. The approval of tirzepatide for moderate-to-severe OSA in adults with obesity represents a milestone, validating the concept that targeting metabolic drivers can yield clinically meaningful improvements in sleep-disordered breathing. Concurrently, combination strategies targeting upper airway neuromuscular function, ventilatory stability, and arousal threshold offer additional options for non-obese patients or those with predominant non-anatomical traits. Gene therapy remains preclinical but illustrates the long-term potential for durable, targeted interventions. Realising the full potential of OSA pharmacotherapy will require rigorous patient phenotyping, robust clinical trial designs that incorporate patient-important outcomes, and the development of integrated care pathways that position drugs alongside, rather than in competition with, established device-based therapies.

From a clinical stratification perspective, the Baveno classification offers a useful framework for identifying patients most likely to benefit from active intervention, by integrating symptom burden and cardiovascular risk alongside AHI. Although not yet validated for guiding specific drug choices, it aligns conceptually with the endotype-driven approach advocated in this review. Future pharmacological trials should consider stratifying patients by Baveno category and other phenotypic traits, to better match therapies to individual risk profiles and ultimately translate the precision medicine vision into routine practice.

## Figures and Tables

**Table 1:** Drugs in clinical trials.

Drug	Mechanism	Clinical Trial Phase	Key Results
Atomoxetine (monotherapy)	Norepinephrine reuptake inhibitor	Phase 2 (completed)	ESS decreased from 15.3 to 10.5 (*p* < 0.01); CGI improved from 4.3 to 3.1 (*p* < 0.01). RDI did not change significantly; REM sleep was reduced.
Desipramine	Norepinephrine reuptake inhibitor	Phase 2 (completed)	Improved Pcrit from −1.9 to −5.2 cmH_2_O and reduced AHI in patients with poor neuromuscular compensation
Reboxetine + Oxybutynin	NRI + antimuscarinic	Phase 2 (completed)	Median AHI reduction 59%; placebo reduction was 6%; PVT median reaction time improved from 250 ms to 223 ms (*p* < 0.001).
AD109 (aroxybutynin + atomoxetine)	Anti-M2 muscarinic + NRI	Phase 3 (ongoing)	MARIPOSA (phase 2b): median AHI reduced 47.1%; 95% CI −61.2 to −27.9; 41% achieved post-treatment AHI < 10 events/h.
Sultiame	Carbonic anhydrase inhibitor	Phase 2 (completed)	Dose-dependent AHI reduction: −16.4% (100 mg), −30.2% (200 mg), −34.6% (300 mg)
Acetazolamide	Carbonic anhydrase inhibitor (reduces high loop gain)	Phase 4/retrospective	Reduces AHI by ~40% in high-loop-gain phenotype
Eszopiclone	Sedative hypnotics (raise arousal threshold)	Phase 2/3	Eszopiclone significantly reduced AHI (MD −5.73 events/h; 95% CI −8.90 to −2.57); zolpidem showed no significant effect
Trazodone	Sedative hypnotic (raises arousal threshold)	Phase 2	Increased the arousal threshold during NREM sleep without worsening upper airway collapsibility.
Zopiclone	Sedative hypnotic (raises arousal threshold)	Phase 2	Increased arousal threshold without impairing genioglossus activity.
Donepezil	Cholinesterase inhibitor (raises arousal threshold)	Phase 2 (completed)	Increased arousal threshold but did not consistently reduce AHI.
Intranasal corticosteroids (fluticasone, budesonide)	Local anti-inflammatory (reduce mucosal edema)	Phase 2/3 (multiple RCTs)	Meta-analysis significant AHI reduction (SMD −0.95, 95% CI −1.42 to −0.47), but with heterogeneity (I^2^ = 62%); In allergic rhinitis patients, AHI decreased from 30.3 to 23.3 (*p* < 0.05).
Liraglutide/Semaglutide	GLP-1 receptor agonists (weight loss)	Phase 3 (completed)	AHI reduction parallel to weight loss
Tirzepatide	GIP/GLP-1 dual agonist	Phase 3 (completed; FDA approved Dec 2024)	SURMOUNT-OSA: Study 1 (non-PAP): AHI reduction ~55% (LS mean change −25.3 events/h; placebo-subtracted −20.0 events/h, 95% CI −25.8 to −14.2; *p* < 0.001); 43.0% achieved remission. Study 2 (on PAP)—AHI reduction ~62.8% (placebo-subtracted −23.8 events/h, 95% CI −29.6 to −17.9; *p* < 0.001); 51.5% achieved remission. Mean body weight reduction 15–20% at 52 weeks.
Retatrutide	GIP/GLP-1/glucagon triple agonist	Phase 3 (ongoing)	AHI change as primary endpoint in nested OSA protocols
Maridebart cafraglutide (AMG 133)	GIPR antagonist + GLP-1 agonist (long-acting)	Phase 3 (ongoing)	
HRS9531	GIP/GLP-1 dual agonist	Phase 3 (ongoing)	
SGLT-2 inhibitors (empagliflozin, dapagliflozin)	Sodium-glucose cotransporter-2 inhibition	Phase 2/3 (meta-analyses; ongoing trial)	

## Data Availability

No new data were created or analyzed in this study. Data sharing is not applicable to this article.

## Abbreviations

The following abbreviations are used in this manuscript:

AAV	Adeno-Associated Viral
AHI	Apnea Hypopnea Index
BL-OG	Bioluminescence Optogenetics
CNO	Clozapine-N-Oxide
CPAP	Continuous Positive Airway Pressure
DREADD	Designer Receptor’s Exclusively Activated by Designer Drugs
FDA	U.S. Food and Drug Administration
GIP	Glucose-Dependent Insulinotropic Polypeptide
GLP-1	Glucagon-Like Peptide-1
HOMA-IR	Homeostatic Model Assessment for Insulin Resistance
hs-CRP	High-Sensitivity C-Reactive Protein
J60	JHU37160-Dihydrochloride
MD	Mean Difference
NBSH	Non-Benzodiazepine Sedative Hypnotics
NRIs	Norepinephrine Reuptake Inhibitors
OAT	Oral Appliance Therapy
OSA	Obstructive Sleep Apnea
PAP	Positive Airway Pressure
Pcrit	Active Pharyngeal Critical Closing Pressure
PVT	Psychomotor Vigilance Task
RCT	Randomised Controlled Trial
RDI	Respiratory Disturbance Index
SGLT-2	Sodium-Glucose Cotransporter-2
T2DM	Type 2 Diabetes Mellitus
